# Complete-arch fixed reconstruction by means of guided surgery and immediate loading: a retrospective clinical study on 12 patients with 1 year of follow-up

**DOI:** 10.1186/s12903-019-0941-z

**Published:** 2020-01-16

**Authors:** Henriette Lerner, Uli Hauschild, Robert Sader, Shahram Ghanaati

**Affiliations:** 1 Baden-Baden, Germany; 2 Department of Oral, Cranio-Maxillofacial and Facial Plastic Surgery, Johann Wolfgang Goethe-University, Frankfurt am Main, Germany; 3 Department of Post-Graduate Medicine, Johann Wolfgang Goethe-University, Frankfurt am Main, Germany; 4 Sanremo, Italy; 5 Department of Oral, Cranio-Maxillofacial and Facial Plastic Surgery, Medical Center of the Goethe University Frankfurt, Frankfurt am Main, Germany; 6 Institute of Pathology, University Medical Center, Johannes Gutenberg University, Mainz, Germany

**Keywords:** Guided implant surgery, Flapless, Immediate implant placement, Immediate loading, Complete-arch reconstructions, Survival, Success

## Abstract

**Background:**

Guided implant surgery is considered as a safe and minimally invasive flapless procedure. However, flapless guided surgery, implant placement in post-extraction sockets and immediate loading of complete-arch fixed reconstructions without artificial gum are still not throughly evaluated. The aim of the present retrospective clinical study was to document the survival and success of complete-arch fixed reconstructions without artificial gum, obtained by means of guided surgery and immediate loading of implants placed also in fresh extraction sockets.

**Methods:**

A total of 12 patients (5 males and 7 females, with a mean age of 50.0 ± 13.8) were enrolled in this study. Implant planning was performed with a guided surgery system (RealGuide®, 3Diemme, Como, Italy), from which 3D-printed surgical templates were fabricated. All implants (Esthetic Line-EL®, C-Tech, Bologna, Italy) were placed through the guides and immediately loaded by means of a temporary fixed full-arch restoration without any artificial gum; the outcome measures were implant stability at placement, implant survival, complications, prosthetic success, soft-tissue stability, and patient satisfaction.

**Results:**

One hundred ten implants (65 of them post-extractive) were placed flapless through a guided surgery procedure and then immediately loaded by means of provisional fixed full arches. Successful implant stability at placement was achieved in all cases. After a provisionalization period of 6 months, 72 fixed prosthetic restorations were delivered. Only 2 implants failed to osseointegrate and had to be removed, in one patient, giving a 1-year implant survival rate of 98.2% (108/110 surviving implants); 8/12 prostheses did not undergo any failure or complication during the entire follow-up period. At the 1-year follow-up control, soft-tissue was stable in all patients and showed satesfactory aesthetic results.

**Conclusions:**

Within the limits of this study, complete-arch fixed reconstruction by means of guided surgery and immediate loading of implants placed in fresh extraction sockets appears to be a reliable and successful procedure. Further long-term prospective studies on a larger sample of patients are needed to confirm these positive outcomes.

## Background

The immediate functional loading of implant-supported, fixed full-arch prostheses can today represent a predictable solution for the rehabilitation of edentulous patients [1–3], even in the case of implant placement in fresh post-extraction sockets [4].

Such procedures as immediate prosthetic loading and immediate placement of implants in fresh extraction sockets are highly appreciated by patients, because they reduce the invasiveness and the number of surgical and prosthetic sessions, as well as the length of time needed for treatment [5, 6].

However, the immediate placement of implants in post-extraction sockets and their immediate functionalization in a complete-arch reconstruction represent a serious challenge for clinicians [2, 4, 6]. Surgically, in fact, clinicians must be able to mentally visualize the future prosthetic rehabilitation and, consequently, the ideal position and axis of implant insertion; in a rather large field such as that of the edentulous maxilla or mandible, this can be particularly complex [2, 4, 6]. Furthermore, it may be difficult to obtain adequate primary stability when placing implants in post-extraction sockets [2, 4, 6, 7]. These mental and clinical difficulties may result in a non-optimal placement of the implants [4, 6].

This non-optimal positioning, even if it does not lead to the violation of anatomical risk structures (such as the inferior alveolar nerve and the maxillary sinus), can have serious aesthetic consequences, and may force the prosthodontist to seek compromise rehabilitative solutions that might not be appreciated by the patient [3–8]. In fact, it is known from the literature how the aesthetics, survival, and long-term success of implant-supported restorations depend not solely on the volume of bone and mucosal tissues available, but also on other parameters, including the implant insertion axis [9–12].

Modern digital technologies [13], in particular guided implant surgery [14–16], now offer a solution to these problems.

Conceived in the mid-nineties, guided implant surgery has rapidly grown in popularity, and is now widely used all over the world [14–16]. The introduction of cone-beam computed tomography (CBCT) allowed the acquisition of the three-dimensional (3D) bone volumes of the jaws in a simple way and with a considerable reduction in the dose of radiation absorbed by the patient, compared to that of conventional computerized tomography [17, 18].

The information on the patient’s bone anatomy, captured by CBCT, may be imported as digital imaging and communication in medicine (DICOM) files into specific software for implant surgery planning and combined with the wax-up of the ideal prosthetic restoration [14–18]. In this software, the surgeon can plan the implant insertion, based on the anatomy of the residual bone and the ideal prosthetic project. According to the planning, a surgical guide is drawn, produced with additive techniques, and used during the implantation for the guided placement of the fixtures [14–19].

In 2002, the concept of guided implant planning linked to immediate functional loading was first introduced in Leuven, Belgium. The first treatments were limited to the edentulous jaws and required full-thickness flaps, as the surgical templates were bone-supported [14–19]. Subsequently, planning procedures have been optimized, opening the way for new types of increasingly precise surgical templates, with mucosal and dental support, to be used in both arches, in partially and completely edentulous subjects [14–19]. The possibility of a flapless approach has further increased the benefits of guided surgery, as this approach reduces the invasiveness and timing of surgical treatment, simplifying the procedure for the clinician and reducing discomfort and morbidity for the patient [20, 21].

However, in most clinical studies in the literature, full-arch restorations are represented by Toronto Bridges, also known as “all-on-four” and “all-on-six” dentures, i.e., hybrid fixed prostheses characterized by the presence of a bar connecting the implants and, more importantly, artificial gum (either in porcelain or in resin, depending by the restorative treatment chosen) [22–25]. There is no doubt that even in this context, guided implant surgery offers advantages, such as more precise implant placement, especially with respect to the screw holes, and the availability to pre-fabricate a milled provisional for same- (or next-) day delivery [14–16, 20, 22–25]. However, these types of implant-supported dentures, although easier to manage for clinicians, cannot represent the maximum of aesthetics, precisely because of the presence of the artificial gum [26, 27]. Moreover, the correct maintenance of daily oral hygiene can be much more difficult for the patient with such types of prosthetic rehabilitation [26, 27].

Today there are few scientific works on complete-arch fixed reconstruction without artificial gum, by means of guided surgery with flapless placement of implants also in post-extraction sockets, and immediate functional loading [28–31].

The aim of the present study is therefore to present the outcome of guided surgery, flapless implant placement, and immediate functional loading of complete-arch fixed prostheses without artificial gum. In particular, we aim to demonstrate how a correct planning of the 3D positioning of the implants allows to obtain complete-arch fixed rehabilitations with highly predictable aesthetics.

## Methods

### Patient selection

A retrospective evaluation was conducted on the customized records of patients that were treated with guided implant surgery during the period from January 2014 to December 2017, in a private dental clinic in Baden-Baden, Germany (Henriette Lerner Dental Clinic).

Patients were included according to the following criteria.

Inclusion criteria:

- Adult and able to provide informed consent;
- Patients in need to tooth extraction and immediate implantation;
- Patients that recieved flapless guided surgery and complete-arch fixed prostheses without artificial gum;
- Patients, that were followed up for at least 1 year.

Exclusion criteria:

- Patients with any other prosthetic rehabilitation than complete-arch fixed prostheses without artificial gum.

The analyzed records included patient-related information (gender, age at surgery, systemic health, smoking habit) details about the inserted implants (type, position, length, and diameter) and the prosthetic rehabilitation (single crown, fixed partial prosthesis, fixed full arch) including the dates of delivery. In addition, the analyzed data included all information about any implant failure or complication that occurred during the intervention, after the surgery, and at each follow-up visit. According to the inclusion and exclusion criteria, only patients treated with guided implant surgery and immediate loading by means of complete-arch fixed reconstruction without artificial gum were considered eligible and thus enrolled in the study. A necessary condition for enrollment in the present study was also the patient’s willingness to present him/herself at a final control visit. All other patients (i.e., patients treated with dental implants to restore partially or totally edentulous jaws, without the aid of guided surgery; or patients treated with guided implant surgery but restored with fixed full-arch maxillary prostheses with artificial gum) were excluded from this study. The principles highlighted in the Helsinki Declaration on experimentation on human subjects were strictly followed. This retrospective study received ethical approval from the Institutional Review Board of the Goethe University of Frankfurt, Germany (number: 182/19). All patients were fully informed on the nature of this retrospective study, read and signed a written consent form for inclusion, and for the analysis of their records, that was approved by the University. In addition, the authors obtained written consent for publication from the patients enrolled in this retrospective study.

### Data acquisition

**Fig. 1:**
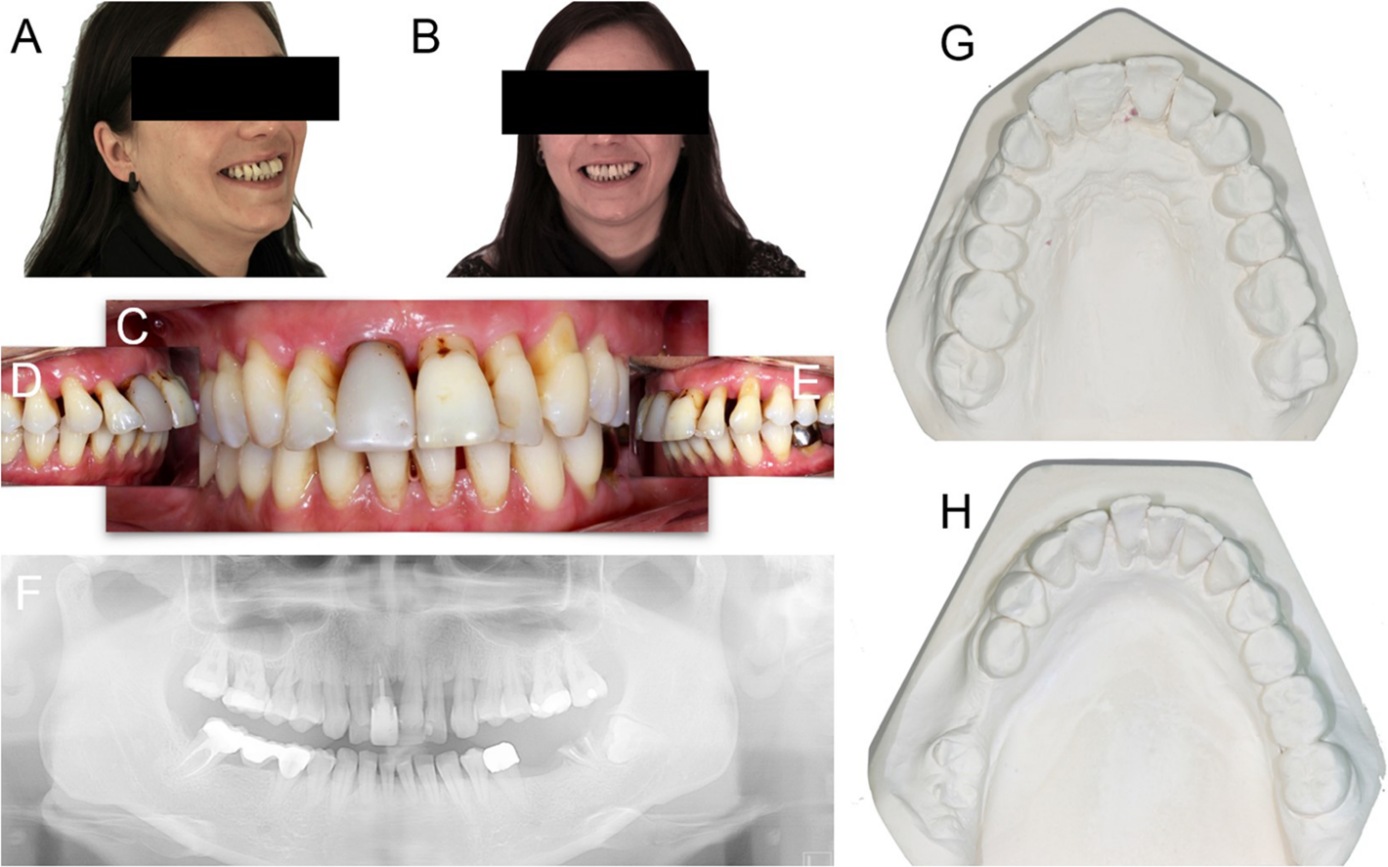
Pre-operative initial situation. Lateral (**a**) and frontal (**b**) view of a 32-year-old female patient, in good systemic health, presented with an advanced chronic periodontitis, teeth mobility and recurrent infections, persisting over years. In the upper jaw, in particular, all teeth were sensitive and elongated and this seriously compromised the function and the aesthetic of the smile. The patient suffered from this situation and she was strongly motivated to solve this biological, functional and aesthetic problem. **c** Frontal and (**d**, **e**) lateral intraoral pictures. **f** Panoramic radiograph. In the maxilla, the severe chronic periodontitis determined an advanced bone loss, that compromised the stability of all teeth. **g** Upper and (**h**) lower jaw precision models

**Fig. 2:**
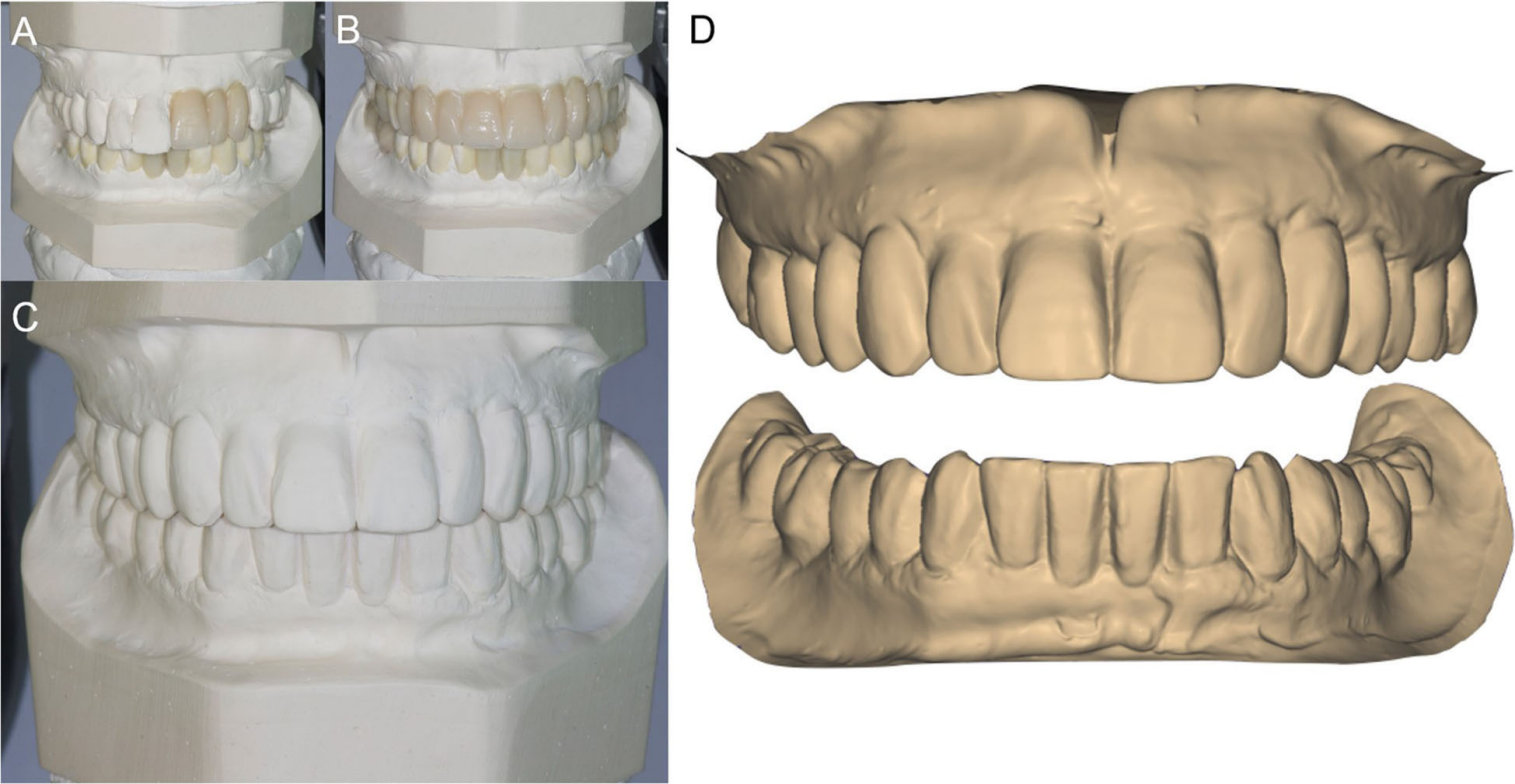
Digital smile design and diagnostic wax-up. **a** Starting from the photographic data, a digital smile design software was used for a first evaluation of the length and width of the teeth. The smile design indicated the need to increase the length and the width of the patient’s teeth, particularly in the anterior maxilla. There was a need to modify the teeth shape as well. **b** The information taken from the smile design was then used for the preparation of a diagnostic wax-up. **c** The models were mounted in articulator and the indications of the smile design were manually replicated in the wax-up. **d** The initial situation, the wax-up and the situation after extractions were then scanned with a desktop scanner (Deluxe®, Open Technologies srl, Brescia, Italy), in order to have all the. STL files available for upload in the guided surgery software

A complete clinical, photographic, and radiographic dataset was acquired for each patient. In particular, photographs of the initial situation were taken (Fig. 1a, b, c, d, e) accompanied by two-dimensional (panoramic) radiographs (Fig. 1f), periodontal probing and three-dimensional radiographs (CBCT of both arches). General impressions were taken, and stone casts were obtained for the study of the case (Fig. 1g, h). Starting from the photographic data, a digital smile-design software was used for a first evaluation of the length and width of the teeth. The information taken from this analysis was used for the preparation of a diagnostic wax-up on the stone cast models (Fig. 2a, b, c). Finally, the initial situation, the diagnostic wax-up (and therefore the ideal morphology of the teeth) and the situation after virtual extractions were then scanned with a desktop scanner (Deluxe®, Open Technologies srl, Brescia, Italy), in order to have all the. STL files available for upload in the guided surgery software (Fig. 2d).

### Digital implant planning and laboratory workflow

**Fig. 3:**
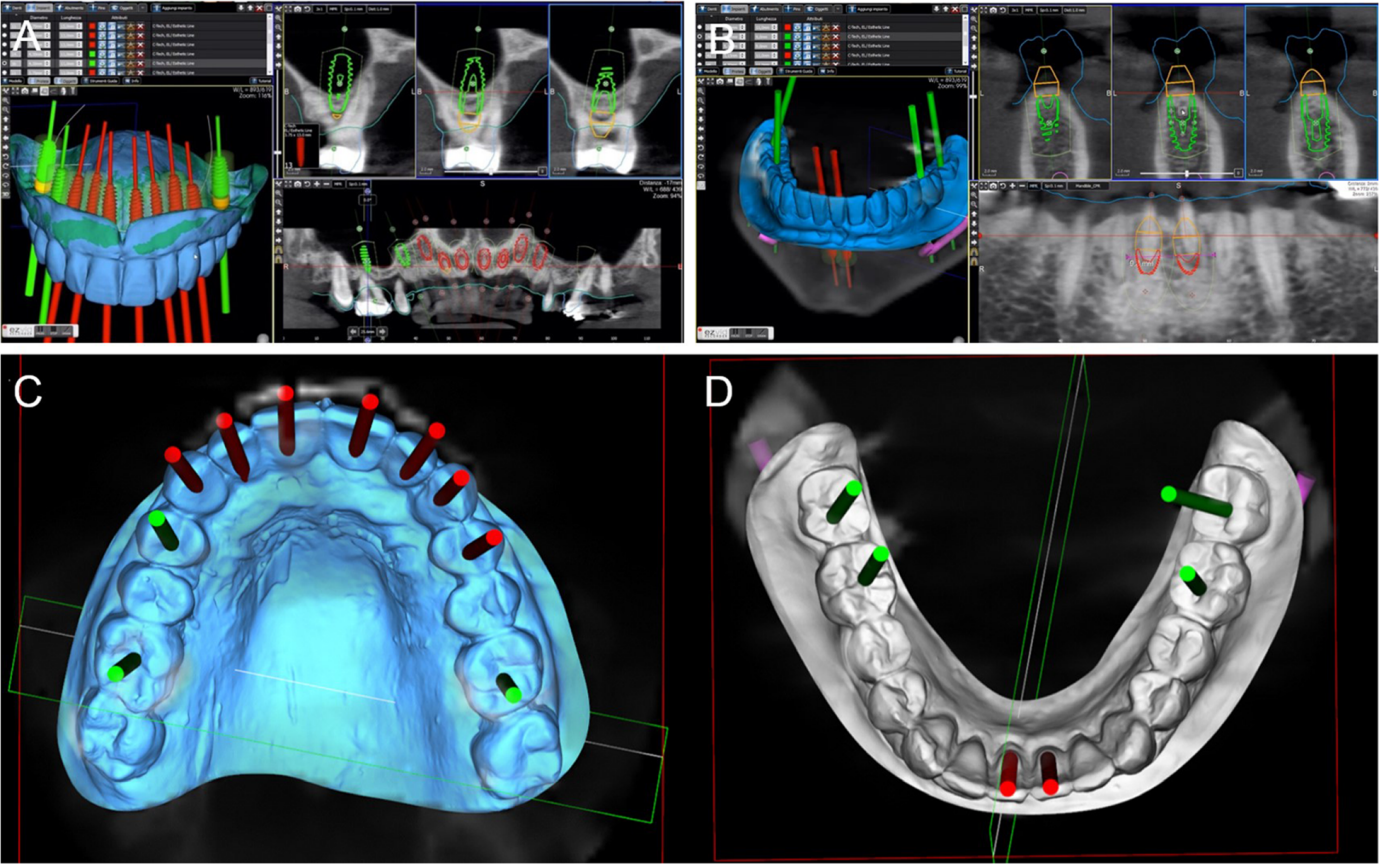
Prosthetically driven 3D implant planning. **a** In a guided surgery software (RealGuide®, 3Diemme, Como, Italy) the. STL file with the ideal teeth morphology (model of the maxilla with included wax-up) was imported and superimposed on the bone anatomy, obtained from the CBCT. Ten immediate post-extraction implants (#16, #14, #13, #12, #11, #21, #22, #23, #24, #26) were planned in the cross sections of the CBCT (placement of #16 and #26 required maxillary sinus augmentation). The implant position, inclination and depth were carefully planned, trying to engage the fixtures as much as possible into the bone, to increase primary implant stability in the fresh extraction sockets, and taking into account the emergence profile and the overlying future prosthesis, so that implants were placed in a prosthetically driven matter. The final prosthetic plan foresaw rehabilitation with single crowns in the frontal area (from #14 to #24) and rehabilitation with partial fixed prosthesis for the posterior sectors (from #15 to #17, and from #25 to #27, respectively). **b** A similar procedure was performed in the mandible, with the superimposition of the. STL file with the ideal teeth morphology on the bone, and therefore the implant planning. Six implants were originally planned (#47, #46, #41, #31, #35, #37) in the cross sections of the CBCT. Once again, the implant position, inclination and depth were carefully planned, trying to engage the fixtures as much as possible into the bone, to increase primary implant stability, and taking into account the emergence profile and the overlying future prosthesis, so that implants were placed in a prosthetically driven position. **c**, **d** Visualization of the implant emergence profiles in relation to the ideal position of the teeth and the prosthetic plan, in the maxilla and mandible. The prosthetic axes of the posterior implants emerged in the masticatory center of each tooth, in the anterior individual zirconia abutments cemented on titanium bases were chosen. On the upper jaw ten implants were planned, on the lower jaw six

**Fig. 4:**
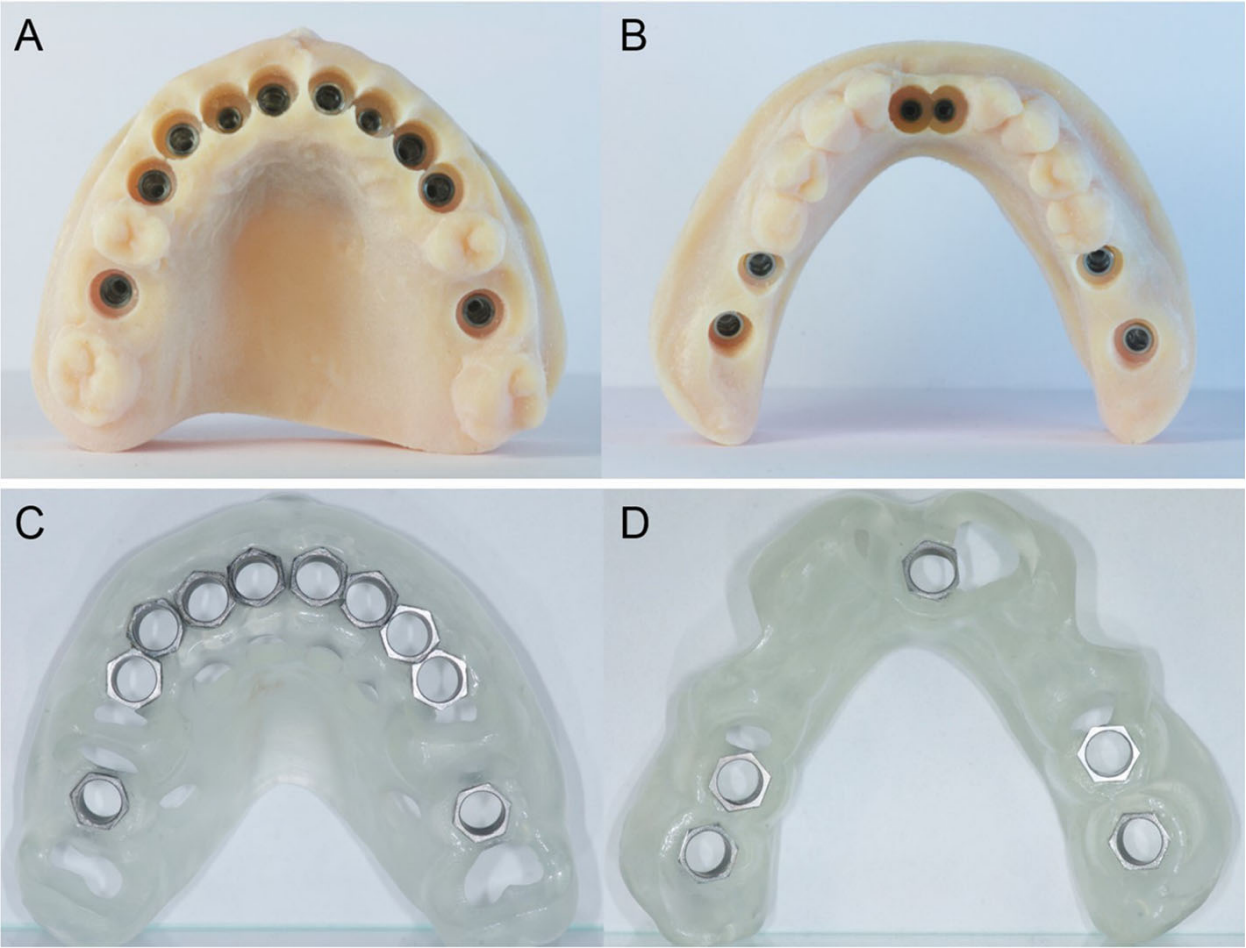
3D printing of the models and the surgical guides. **a**, **b** The models were printed in the laboratory with a 3D printer (Form2®, Formlabs Inc., Somerville, MA, USA) and the implant analogues were positioned. These models not only included the correct position of the planned implants, but also the mucosa and the residual teeth, that have not been removed in the planning, in order to facilitate the superimposition in the guided surgery software, and to stabilize the surgical guide during the intervention. The compromised teeth had to be subsequently removed during surgery. **c**, **d** 3D printing of the models and the surgical guides. Based on the virtual planning, surgical guides were printed too. The sleeves were then inserted in the guides. In the lower jaw, since the sleeves of the incisors touched each other, two different guides were 3D printed

**Fig. 5:**
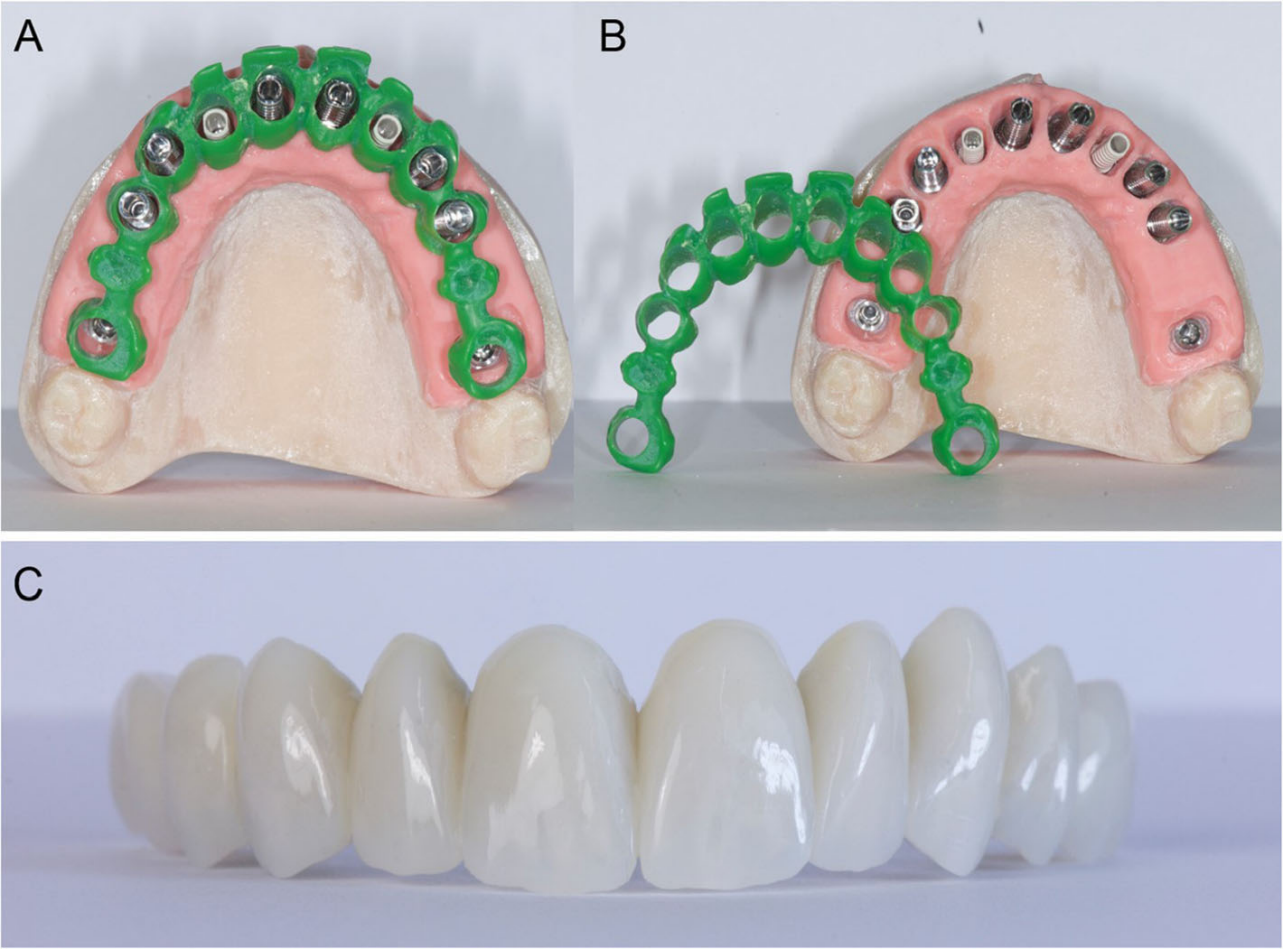
Preparation of the temporary fixed full arch for immediate loading. **a**, **b** After CAD design, a burn-out framework based on the prosthetic project and virtual implant position was milled. **c** This led to a metal structure on which resin composite was manually stratified, in order to obtain a highly aesthetic temporary full-arch prosthesis for immediate loading

In a guided surgery software, the. STL files with the ideal teeth morphology, derived from the scan of the diagnostic wax-up, were imported and superimposed on the bone anatomy, obtained from the CBCT. Then, the surgeon (H.L.) and the dental technician (U.H.) were able to plan the placement of the implants in the correct position, depth, and inclination, guided by the prosthetic wax-up, in a prosthetically-driven manner (Fig. 3a, b, c, d). Care was taken to try to engage the fixtures as much as possible in the residual bone, exceeding the apex of fresh extraction sockets at least 3–4 mm, by choosing appropriate implant length. At the same time, care was taken to position the implants in the palatal portion of the sockets, ideally at a distance of 2–3 mm from the residual buccal bone walls, and at a proper inclination. Ideally the axis of the implants had to be in the center of the teeth, to achive a perfect prosthetic plan. The same procedure was repeated in both maxilla and mandible. After the planning was successfully completed and carefully controlled, the models of the situation were 3D printed in the laboratory with a desktop printer and the implant analogues were inserted in these models (Fig. 4a, b). These models not only included the correct position of the planned implants, but also the mucosa and the residual teeth, which had not been removed in the planning, in order to facilitate the superimposition in the guided surgery software and to stabilize the surgical guide during the intervention (these hopeless teeth had to be removed at the end of the intervention). Based on the virtual planning, surgical guides were then printed, and the sleeves were manually inserted in (Fig. 4c, d). In the lower jaw, since the sleeves of the central incisors touched each other, two different guides were 3D printed. Finally, before surgery, provisional full-arch restorations were prepared for immediate loading and aesthetics. A burn-out framework based on the prosthetic project and virtual implant position was milled (Fig. 5a, b). This led to a metal structure on which resin composite (Nexco®, Ivoclar Vivadent, Schaan, Liechtestein) was manually stratified, in order to obtain a highly aesthetic temporary full-arch prosthesis for immediate loading (Fig. 5c). This prosthesis had to be seated on temporary abutments immediately after implant placement.

### Implant type

The implants used in this study (Esthetic Line-EL®, C-Tech, Bologna, Italy) were conical, with a Morse-taper hexed connection and an acid-etched surface. In details, these implants incorporate three different threading profiles, designed to adapt to the different bone structures that occurr along the depth of the fixture. In the coronal portion, in fact, microgrooving is present, whereas in the intermediate portion, the fixtures present a double lead thread, to facilitate insertion and to increase primary stability, particularly in soft bone. In the apical portion, these implants are endowed with aggressive apical threading which is particularly indicated to stabilize the fixture in post-extraction sockets, in the case of immediate placement after extraction; however, a rounded apex protects such anatomical structures as the maxillary sinus membrane and inferior alveolar nerve. Further characteristics of these implants are a beveled shoulder, to facilitate bone growth in the case of subcrestal placement in post-extraction sockets; a Morse-taper hexed connection, to reduce screw loosening and micromovements of the abutment; and a concave aesthetic concept with platform switching, for better soft-tissue healing. The presence of one connection for all implant diameters facilitates the prosthetic treatment, by offering a large range of tissue-shaping possibilities. The fixtures used in this study were available in different lengths (8,9,10,11,12,13, and 14 mm) and diameters (3.8, 4.3, 5.1, 6, and 7 mm).

### Surgical and prosthetic treatment

**Fig. 6:**
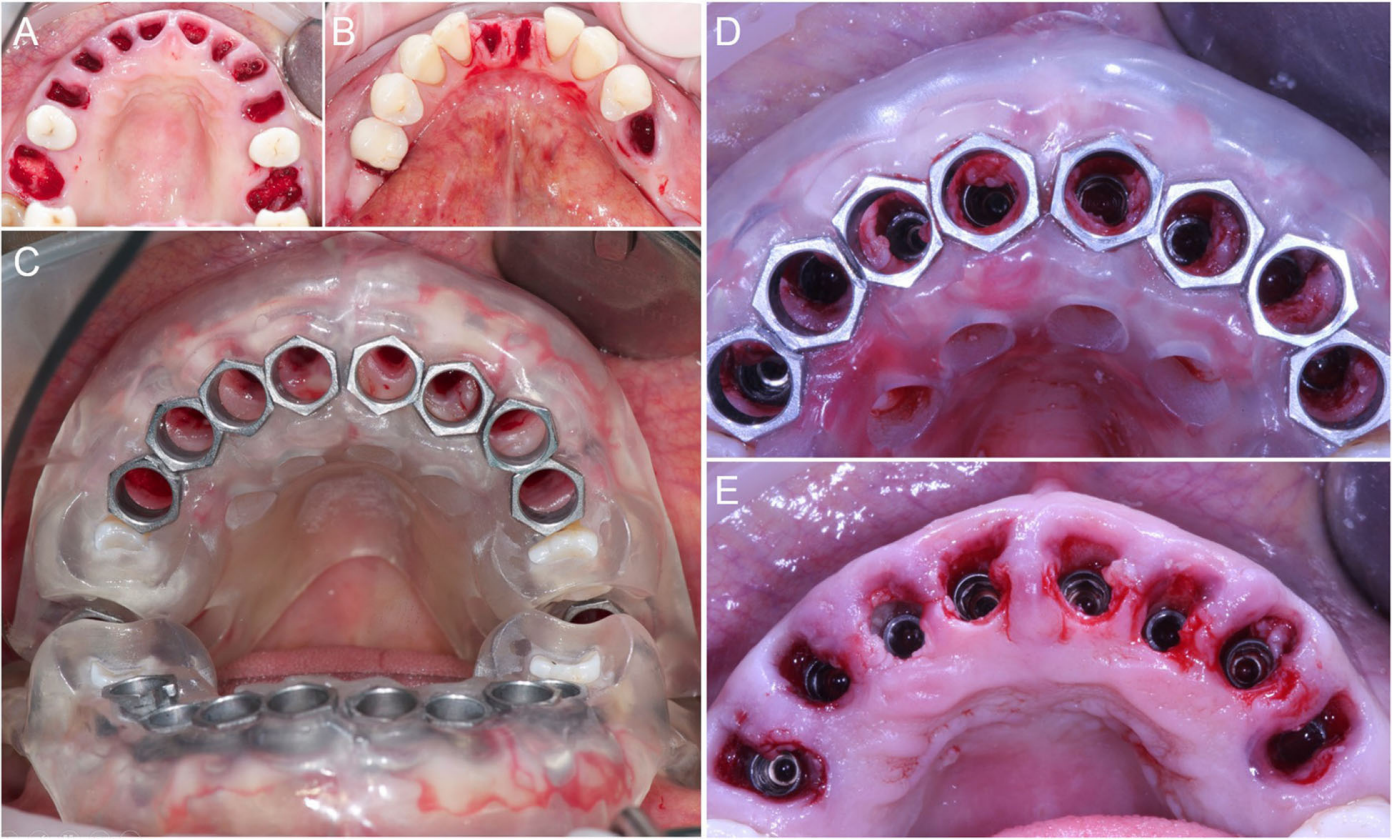
Extraction of the compromised teeth and guided implant surgery. **a** Ten compromised teeth were extracted in the maxilla and (**b**) five teeth in the mandible. **c** Superior guide in position. The guide was stabilized and supported by the teeth #15, #25, the hard tissues of the maxillary ridge and palate. **d** The surgeon performed the implantation through the guide, for a fully guided procedure, so that an aesthetically driven positioning of the implants was achieved. **e** After removal of the guide, it is evident how buccal tissues have been preserved, in order to achieve a highly predictable aesthetic outcome

**Fig. 7:**
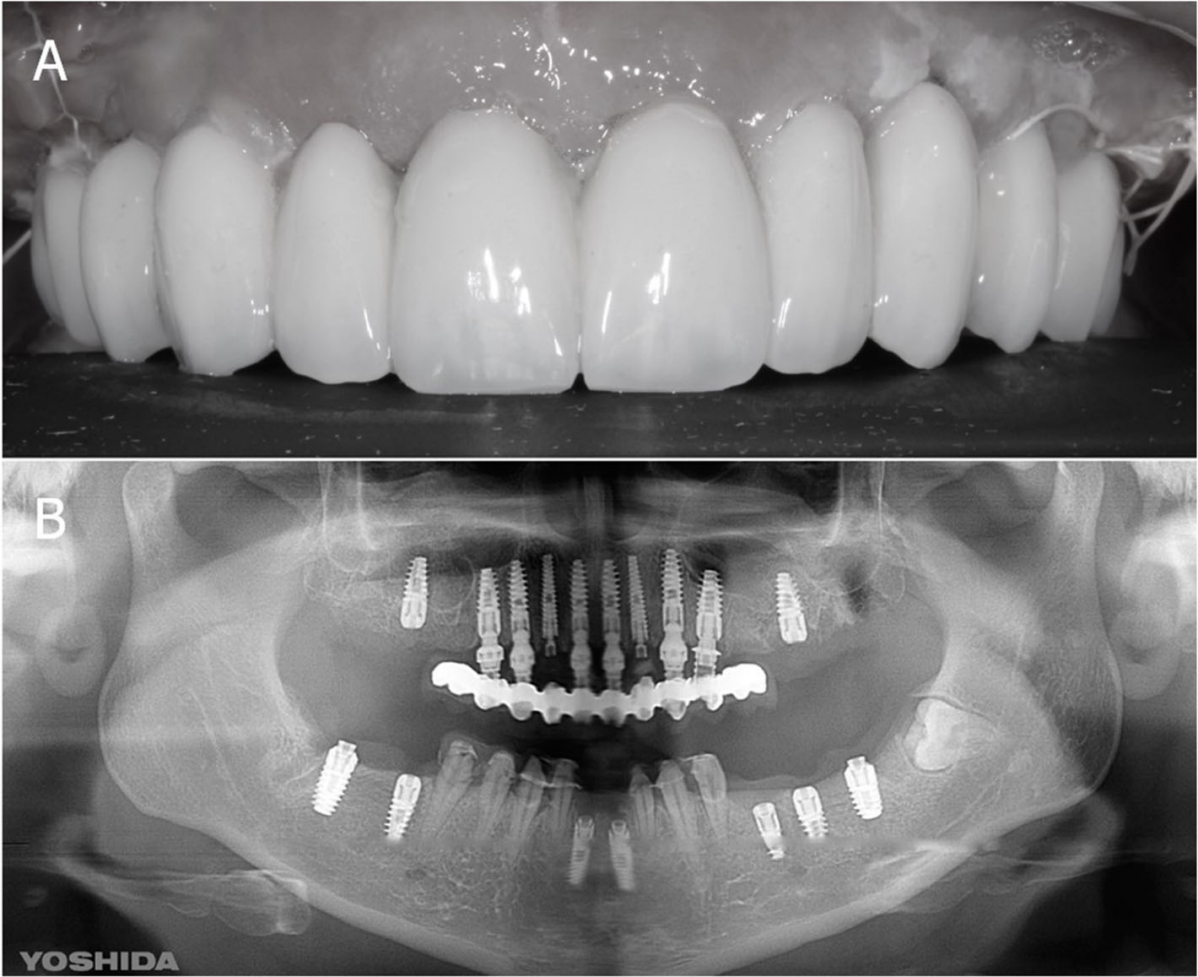
Immediate loading and panoramic control radiograph. **a** Temporary Prosthesis in position 3 days after surgery. **b** Post-operative situation: panoramic radiograph. During surgery, one more mandibular tooth had to be extracted and replaced by an implant

**Fig. 8:**
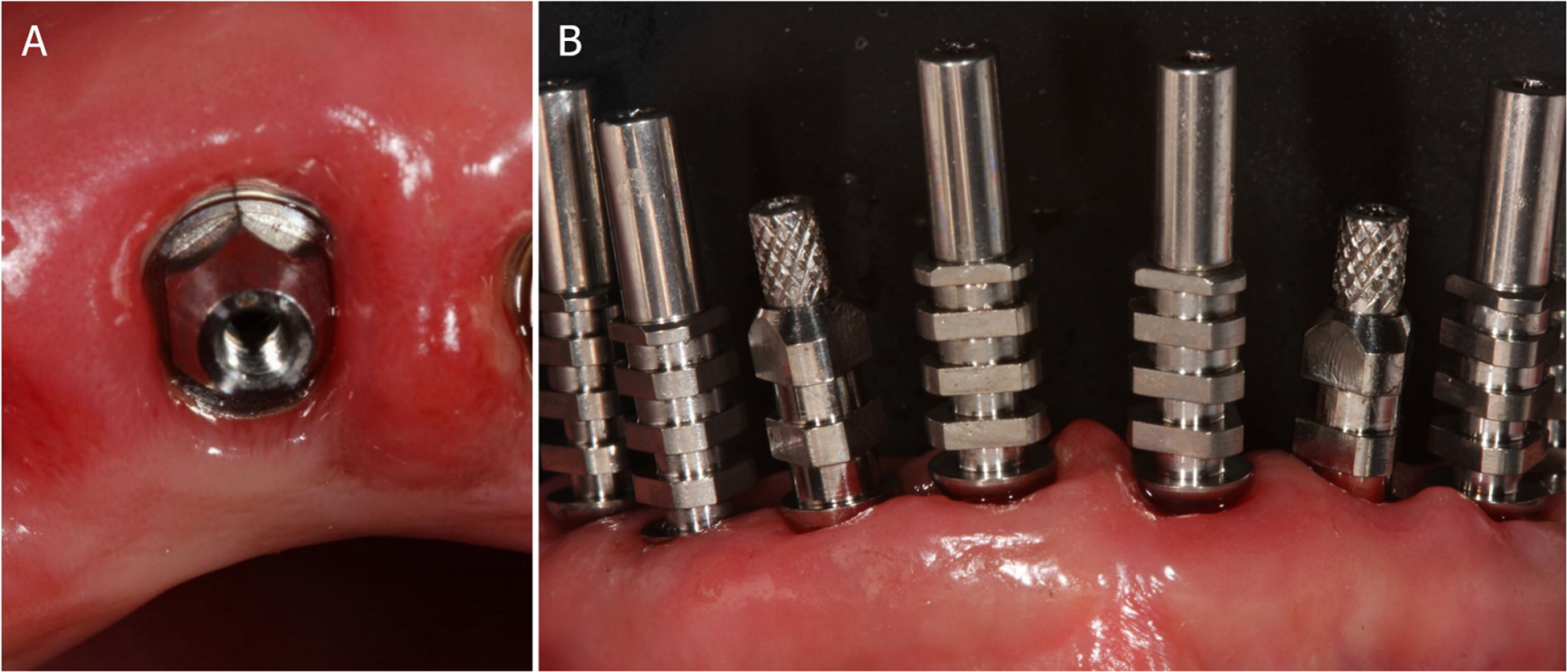
Final intraoral impressions. **a** Impression taking with open tray over multi-unit intermediary abutments, to avoid the damaging of the peri-implant structures. **b** This analogic procedure was considered at the time the most precise for full arch

**Fig. 9:**
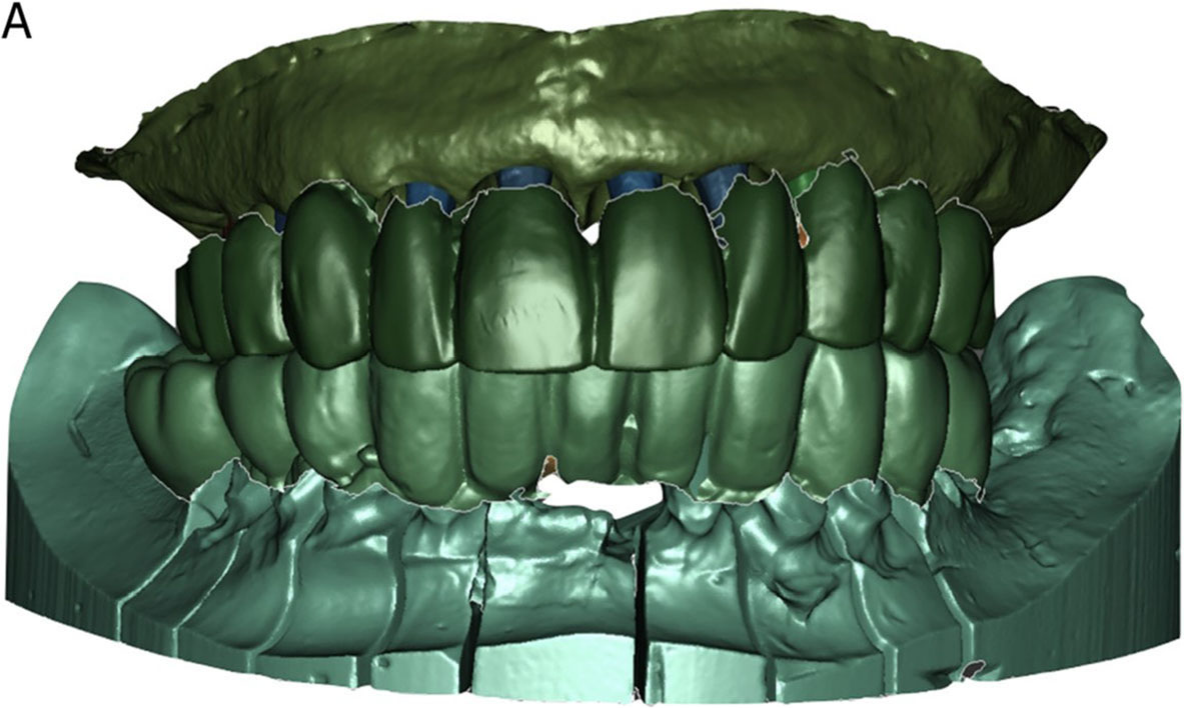
Aesthetic refinement. **a** the clinician took an alginate impression of the provisionals in place, and a precision impression in polyether with open tray, to capture the position of the implants. These impressions were sent to the dental technician who poured plaster models and scanned them. These models were then overlapped in a CAD software, in order to have a guide for modeling the final restorations and to perform aesthetic modifications. All these modifications, however, were done manually, on 3D printed models. Then, the final shapes were scanned again

**Fig. 10:**
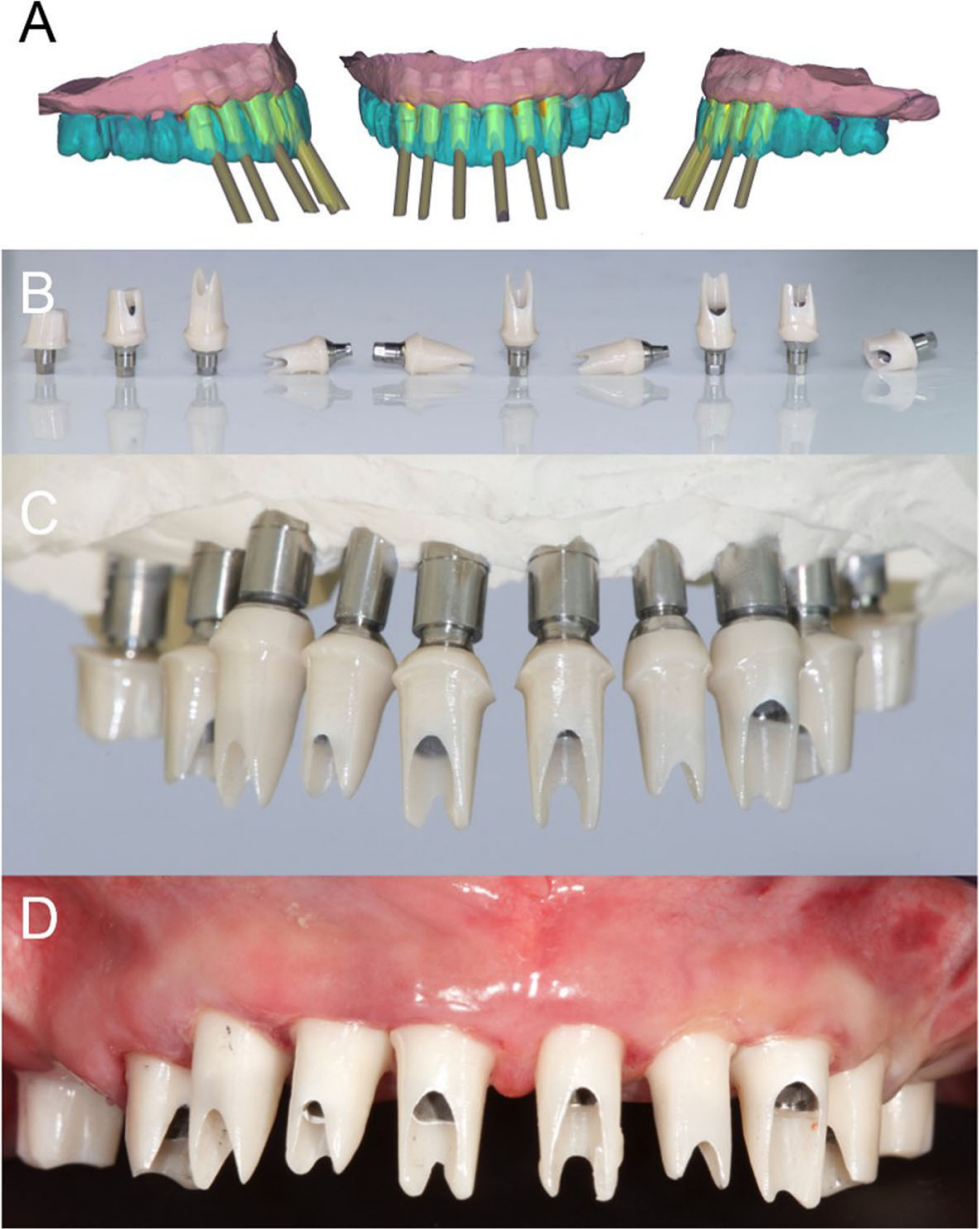
Final CAD/CAM procedures and clinical application. **a** CAD design of individual zirconia abutments. These abutments were designed for extraoral cementation on titanium bases. **b** The milled zirconia abutments glued on titanium bases. **c** The milled zirconia abutments placed on the model. **d** The milled zirconia abutments placed in the patient’s mouth. The abutment margins were planned 0.5 mm below the gingival margin

**Fig. 11:**
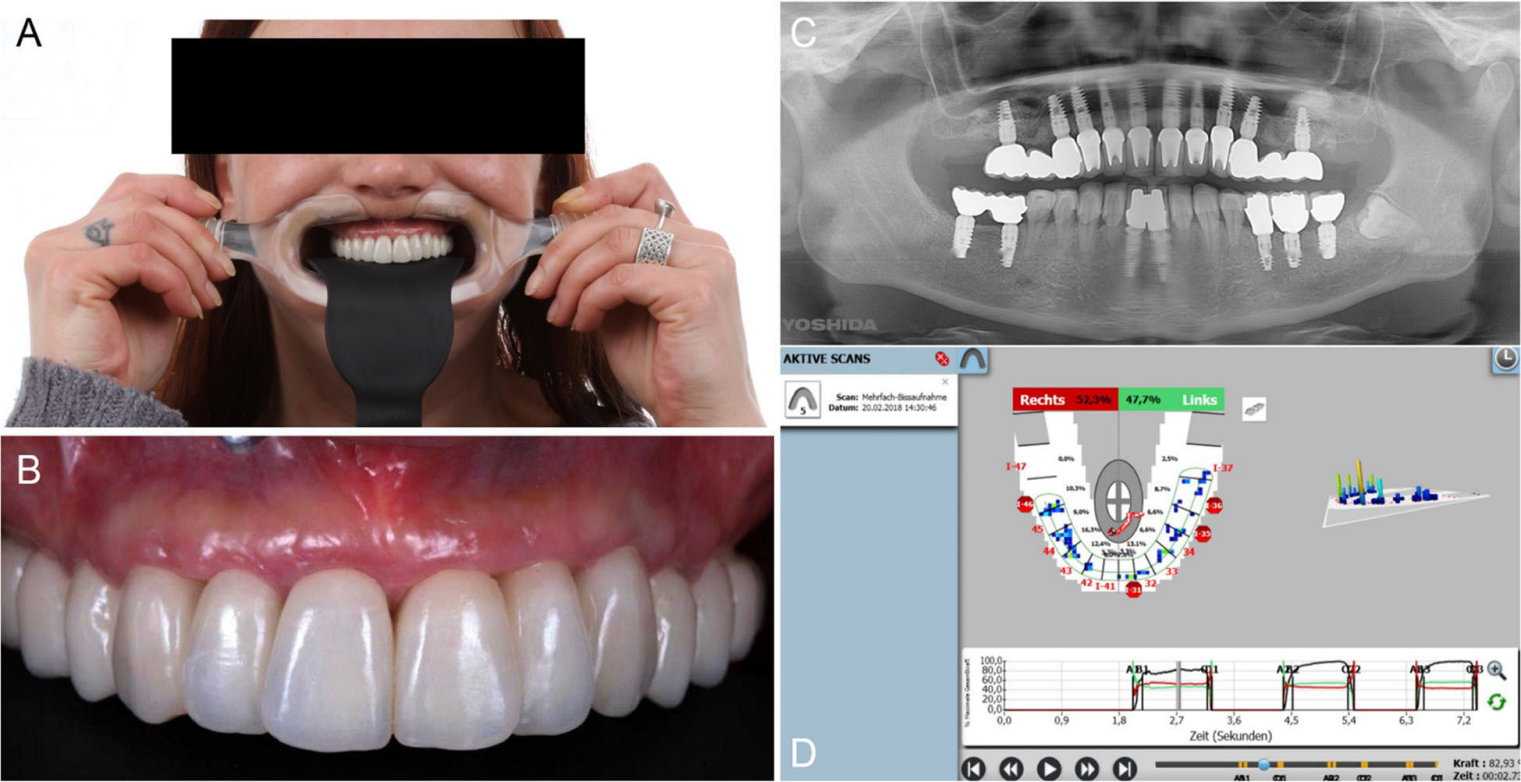
Delivery of the final restorations. **a** Healthy and aesthetic aspect of the restorations. **b** Intraoral picture. **c** Final panoramic radiograph. **d** Occlusal control with Tekscan®. The stability of the occlusal situation was checked after delivery and in the recall sessions, at least once a year

After this, flapless surgery could start with the atraumatic extraction of all hopeless teeth (with the exception of the ones used to stabilize the guide; those teeth had to be removed after the removal of the guide). During extraction, care was taken not to damage the alveoli and particularly the delicate maxillary buccal bone wall (Fig. 6a, b). After the digital planning, the surgical template was positioned, the fit was carefully checked (Fig. 6c), and the guided surgery started with the preparation of all implant sites, using drills of incremental diameter, and ended with the placement of all planned implants, through the guide (Fig. 6d, e). After implant placement, the surgeon could verify the positioning as well as the soft-tissue status and proceed with simultaneos minor/major grafting procedures, where needed. The need for grafting procedures obviously forced the clinician to raise a full-thickness flap, but this procedure was limited exclusively to the area or areas that required bone augmentation; in all other cases and in all other areas, a flapless surgery was carried out. After the surgery was completed, the temporary fixed full-arch provisional in resin with metal framework was carefully adapted and relined on temporary abutments, removed, accurately polished, and then delivered to the patient (Fig. 7a). This fixed full-arch temporary was cemented and a panoramic radiograph was taken (Fig. 7b). Six months after surgery, when the period of provisionalization ended, the clinician recalled the patient and took two different impressions: (i) generic alginate impressions with the provisional prostheses in position; and (ii) precision impression with polyether open tray over multi-unit intermediary abutments, to avoid damaging the peri-implant structures (Fig. 8a, b). These two impressions were sent to the dental laboratory, where plaster casts were poured and scanned with the same aforementioned desktop scanner. These models were then overlapped in a computer-assisted-design (CAD) software, in order to have a guide for modeling the final restorations and performing aesthetic modifications (Fig. 9a). All these modifications, however, were done manually, on 3D-printed models (Fig. 9b, c). Then the final shapes were scanned again. At this point, the dental technician could proceed to design, in a CAD software (Exocad®, Darmstadt, Germany), the final individual, customized abutments (which had to be cemented extraorally on selected titanium bases) and the final restorations (Fig. 10a, b, c). Both the individual, customized abutments and the final restorations were full-ceramic and milled in zirconia with a powerful milling machine (M1 WET Heavy Metal®, Zirkonzhan, Bolzano, Italy), in order to improve the aesthetic outcome. The restorations had to be single crowns (in the frontal area, for a better biological response, with physiological auto-detersion, and to allow for excellent oral hygiene maneuvers, such as the passage of dental floss) and fixed partial prostheses (in the posterior areas, to improve the functional response). At the delivery of the final restorations, the customized abutments were screwed in the correct position: the margins were positioned subgingivally, in accordance with the original surgical and prosthetic plan (Fig. 10d) and all restorations were placed. The marginal adaptation was checked clinically using magnifying glasses (Zeiss 4.5x®, Zeiss, Oberkochen, Germany) and the occlusion was carefully evaluated using articulating papers (Bausch Articulating Paper®, Bausch Inc., Nashua, NH, USA) as well as digital devices (Tekscan®, Boston, MA, USA) prior to cementation. Clinical pictures (Fig. 11a, b) and a panoramic radiograph (Fig. 11c) were taken, along with a digital analysis of the occlusion (Fig. 11d).

### Outcome measures

The outcome measures for the present study were implant stability at placement, implant survival, complications, prosthetic success, marginal bone remodeling, soft-tissue stability, and patient satisfaction.

### Implant stability at placement

Implant stability was measured at placement. Insertion torque (IT) and resonance frequence analysis (RFA) were used as methods for measuring implant stability, as previously described [32, 33]. IT was measured before the removal of the surgical guide. Since all implants were placed using the implant motor, a standard IT of 50 Ncm was set at placement. If the machine-driven insertion was discontinued because of high IT (> 50 Ncm), the implant insertion was completed manually, through the guide, with a dedicated wrench. Conversely, if the final IT was < 45 Ncm, the full procedure was guided by the implant motor. The IT was considered poor when < 35 Ncm. Finally, RFA was used to confirm the stability of each implant, immediately after implant placement, after the removal of the guide. For each implant, the average value from 4 different measurements (buccal, palatal, mesial, and distal) was obtained. The stability of the fixture was considered acceptable with an implant stability quotient (iSQ) ranging from 55 to 85, low with an iSQ < 55. Regardless of the stability value that emerged, all implants were subjected to immediate functional loading, splinted through a temporary full-arch restoration.

### Implant survival

An implant was classified as “surviving” if still functioning regularly at the 1-year follow-up control. Conversely, in all cases in which the implant failed and had to be removed, it was classified as “failed.” The reasons for implant failure were:

1. mobility due to lack of osseointegration, in the absence of clinical symptoms/signs of infection (pain, suppuration, exudation), during the healing period (i.e., the period of provisionalization) or after the delivery of the final restoration;
2. infection with pain, suppuration, exudation, related bone loss (peri-implantitis), and implant loosening;
3. progressive marginal bone loss due to occlusal overloading, in the absence of clinical symptoms/signs of infection (pain, suppuration, exudation);
4. fracture of the fixture.

### Complications

The assessment of immediate operative/ post-operative, biologic, and prosthetic complications included identification of any problem or complication that had affected the guided implant procedures and the implant-supported restorations, from the initial surgery until the end of the 1-year follow-up period. Complications were divided into immediate operative / post-operative (related to the guided surgery procedure), biologic, and prosthetic. Biologic and prosthetic complications could be early or late, according to when they occurred: a complication was defined as early if it occurred no later than the third month after implant placement; conversely, a complication was late if it occurred more than 3 months from implant placement.

### Immediate operative/ post-operative complications were

1. bad adaptation of the surgical guide, i.e., a non-perfect adaptation of the surgical guide when positioning and fixing it;
2. fracture of the surgical guide during surgery, including partial and incomplete fractures of the template structure, which occurred during the preparation of the surgical sites and the positioning of the implants;
3. insufficient implant stability at the removal of the surgical guide;
4. aberrant implant position with buccal bone dehiscence;
5. intra-operative bleeding;
6. lack of passive fitting of the immediate resin prosthesis.

### Biologic complications were

1. post-operative pain and/or swelling;
2. peri-implant mucositis, i.e., superficial inflammation of the peri-implant tissues with involvement of the soft tissues only, characterised by mild discomfort, swelling, and gingival reddening, in the absence of any radiographic evidence of marginal bone loss;
3. peri-implantitis, i.e., deep infection of the peri-implant tissues with involvement of the bone tissue, characterised by pain or discomfort on occlusion, suppuration/exudation, abscess, fistula formation, and/or advanced marginal bone loss (≥2.5 mm);
4. progressive marginal bone resorption in the absence of any kind of infection, i.e., a radiographic peri-implant bone loss > 1.5 mm after the first year of function, and/or exceeding 0.2 mm each following year.

### Finally, prosthetic complications were

(1) mechanical complications, i.e., complications that occurred on pre-formed components and part of the implant system, such as unscrewing of the connecting screw and loss of connection between the implant and the abutment (abutment screw loosening), fracture of the connecting screw (abutment screw fracture), or fracture of the prosthetic abutment;

(2) technical complications, i.e., complications on prosthetic parts designed and built by the dental technician, such as customized zirconia individual abutments and prosthetic restorations (whether temporary or definitive). Among these complications were fracture of the individual abutments as well as fracture or chipping of the prosthetic restorations.

### Prosthetic success

An implant-supported prosthetic restoration was considered successful if it did not present any failure or complication, either of a biological or a prosthetic nature, throughout the entire course of the study, i.e., from the moment of placement with the immediate functional load of the temporary restoration, until replacement with the definitive restoration, and for the whole period in which the definitive restoration remained in situ, from delivery up to the 1-year follow-up control. The possible complications that could occur were the aforementioned procedural, biological, and prosthetic complications, which could affect the implants and/or the prosthetic structure.

### Soft-tissue stability

The soft-tissue stability was verified from clinical photographs, taken at the placement of the definitive restoration and at the 1-year follow-up, with the same digital camera (d7100®, Nikon, Tokyo, Japan) under the same settings (distance 0.6 m, f 22). Only the area of the smile, from the first right premolar to the first left premolar was investigated. The authors compared the stability of the tissues on these frontal pictures, focusing their attention on the stability of the papillae and the soft-tissue contours, using a novel index modified from Furhauser [34].

### Patient satisfaction

At the 1-year follow-up control, each patient was asked to fill out a patient satisfaction questionnaire consisting of 4 different questions. The questions read as follows:

1. Overall, how satisfied are you with the treatment received?
2. Are you satisfied with the function of your implant-supported restorations?
3. Are you satisfied with the aesthetics of your implant-supported restorations?
4. Are you satisfied with the cleanability of your implant-supported restorations?

For each question, the patient could give 5 possible replies: a) very satisfied; b) satisfied; c) neither satisfied nor dissatisfied; d) dissatisfied; e) very dissatisfied.

### Statistical evaluation

All patients’ data were rigorously collected during the study and entered on a spreadsheet for statistical analysis (Excel 2003; Microsoft, Redmond, WA, USA). Descriptive statistics were used to describe the distribution of patients, implants, and restorations. For all qualitative variables (gender, systemic health, smoking habit, distribution of implants per site, position, length, diameter, distribution of restorations), values were expressed in absolute terms and in percentages (%); then, homogeneity or non-homogeneity in the distribution of patients (per gender, age classes, systemic health conditions, and smoking habit), implants (per site and position, length, and diameter), and restorations (per site and type) was calculated using the chi-square test (a statistically significant difference was reported with *p* < 0.05). For quantitative variables (age at surgery), the means, standard deviations (SD), medians, ranges, and 95% confidence intervals (CI) were calculated. The incidence of implant failures and complications was calculted and expressed in absolute values and in percentages (%). Implant survival was calculated at both the implant level and the patient level; with the patient as the statistical unit, he/she was classified as failure even if only one implant failure occurred. Similarly, the prosthetic success was calculated at both the restoration level and the patient level; with the patient as statistical unit, the presence of even a single complication (biologic or prosthetic) determined the allocation of the patient into the category of failure.

## Results

### Patient population, implant distribution, and prostheses

A total of 12 patients (5 males and 7 females, mean age 50.0 ± 13.8 years, median age 54.5 years, CI 95% 42.2–57.9 years) were enrolled in the present retrospective clinical study; 110 implants (65 of them post-extractive) were installed, immediately loaded by means of a provisional fixed full arch. After 6 months of provisionalization, then, 72 fixed prosthetic restorations (53 single crowns, 17 bridges, and 2 fixed full arches) were delivered, in order to prosthetically reconstruct the complete arch. The distribution of patients is summarized in Table 1. The patients’ groups were homogeneous in distribution according to gender (*p* = 0.563) and age class (*p* = 0.738), whereas most of the patients were systemically healthy (*p* = 0.049, with only 2 patients with diabetes mellitus and 2 others with auto-immune diseases) and non-smokers (*p* = 0.008, with only 3 smoking patients). The distribution of the implants is summarized in Table 2. Most of the implants were placed in the maxilla (*p* < 0.001), but the distribution of the fixtures was homogeneous per position (*p* = 0.735). However, the distribution of the fixtures was non-homogeneous per length (p < 0.001, with most of the placed fixtures being 11 or 13 mm in lenght) and per diameter (*p* = 0.001, most of the installed fixtures being 4.3, 5.1, and 6 mm in diameter). Finally, the distribution of the prosthetic restorations is summarized in Table 3. Most of the 72 fixed restorations (45) were delivered in the maxilla, versus only 27 delivered in the mandible, and there was a statistically significant difference in the number of restorations between maxilla and mandible (*p* = 0.033), with the groups that were considered non-homogeneous. Similarly, most of the restorations delivered in this retrospective clinical study were single-crown (53) or short-span restorations (17); thus there was a statistically significant difference in the distribution of the restorations, per type (*p* < 0.001).

**Table 1:** Patient distribution

	N°	Percentage (%)	p*
Overall	12	100%	-
Gender			
Males	5	41.7%	0.563
Females	7	58.3%	
Age at surgery			
20-39	4	33.3%	0.778
40-59	5	41.7%	
60-79	3	25.0%	
Systemic health			
No systemic diseases	8	66.6%	0.049
Diabetes mellitus	2	16.7%	
Immunological disorders	2	16.7%	
Smoking habit			
Non-smokers	9	75.0%	0.008
Light smokers (<10 cigarettes/day)	2	16.7%	
Heavy smokers (≥ 10 cigarettes/day)	1	8.3%	

* *p* = χ^2^ test. A statistically significant difference in the distribution of patients was set with *p* < 0.05

**Table 2:** Implant distribution

	N°	Percentage (%)	p*
Overall	110	100%	-
Site			
Maxilla	75	68.2%	<0.001
Mandible	35	31.8%	
Position			
Incisors	24	21.8%	0.735
Cuspids	26	23.6%	
Premolars	28	25.5%	
Molars	32	29.1%	
Length			
8 mm	10	9.1%	<0.001
9 mm	13	11.8%	
10 mm	12	10.9%	
11 mm	32	29.1%	
12 mm	9	8.2%	
13 mm	23	20.9%	
14 mm	4	3.6%	
15 mm	7	6.4%	
Diameter			
3.8 mm	16	14.5%	0.001
4.3 mm	31	28.2%	
5.1 mm	28	25.5%	
6 mm	27	24.5%	
7 mm	8	7.3%	

* *p* = χ^2^ test. A statistically significant difference in the distribution of patients was set with *p* < 0.05

**Table 3:** Prosthetic restoration distribution

	N°	Percentage (%)	p*
Overall		100%	-
Site			
Maxilla	45	62.5%	0.033
Mandible	27	37.5%	
Type			
Single crowns	53	73.6%	<0.001
Two-units fixed partial prostheses	10	13.9%	
Three-units fixed partial prostheses	7	9.7%	
Full-arch prostheses	2	2.8%	

* *p* = χ^2^ test. A statistically significant difference in the distribution of patients was set with *p* < 0.05

### Implant stability

The mean implant stability at placement, as measured by IT and RFA (iSQ), is summarized in Table 4.

**Table 4:** Distribution of the insertion torque (IT) and implant stability quotient (ISQ) measured at placement

	IT at placement < 35 Ncm (%)	IT at placement ≥ 35 Ncm (%)	ISQ at placement < 55 (%)	ISQ at placement 55<×<85 (%)
Overall	25/110	85/110	28/110	82/110
Maxilla	20/75	55/75	21/75	54/75
Mandible	5/35	30/35	7/35	28/35

### Implant survival

Two implants failed and had to be removed, in the same 57-year-old diabetic female patient. The patient was a light smoker. These implants were lost in the posterior maxilla because they failed to osseointegrate 1 month after surgery and before the final restorations were delivered. Thus, the 1-year implant-based survival rate was 98.2% (108/110 surviving implants). Considering the patient as a statistical unit, with one patient experiencing implant failure and therefore categorized as failure, the 1-year patient-based survival rate was 91.6% (11/12 patients not having any implant failure).

### Complications

During guided surgery procedures, no complications were reported. All surgical guides showed an excellent fit and were sufficiently resistant not to break during the insertion of the fixtures. All implants were sufficiently stable at the removal of the guides and they appeared to be in the planned position, without any evident mistake or aberrant positioning. The immediate provisional fixed full arch was easily adapted on the fixture after implant placement. In addition, over the 1-year follow-up period, no major biological complications affected the surviving (108/110) implants. Two patients had peri-implant mucosal inflammation with bleeding on probing around two post-extraction implants after 3 months, but the improved oral hygiene reduced the inflammation. No peri-implantitis occurred, nor progressive marginal bone resorption. The incidence of biologic complications amounted to 1.8% (2/108 implants). No issues were registered with the provisional restorations. With regard to the final implant-supported fixed restorations, only two single crowns underwent abutment screw loosening; these abutments were re-screwed and no further loosening was reported. The incidence of mechanical complications was therefore 1.8% (2/108) implants. No technical complications were reported.

### Prosthetic success

Considering the implant failures that occurred and the different biologic and prosthetic complications that were encountered, the 1-year prosthetic success of this study amounted to 66.6% (8/12 prostheses did not undergo any failure or complication).

### Soft-tissue stability

The photographic analysis revealed little or no differences between the pictures taken at the delivery of the final restorations and those at the 1-year controls. A certain degree of maturation of soft tissues was also evidenced, with growth of inter-implant papillae and adequate stability of soft-tissue contours.

### Patient satisfaction

**Fig. 12:**
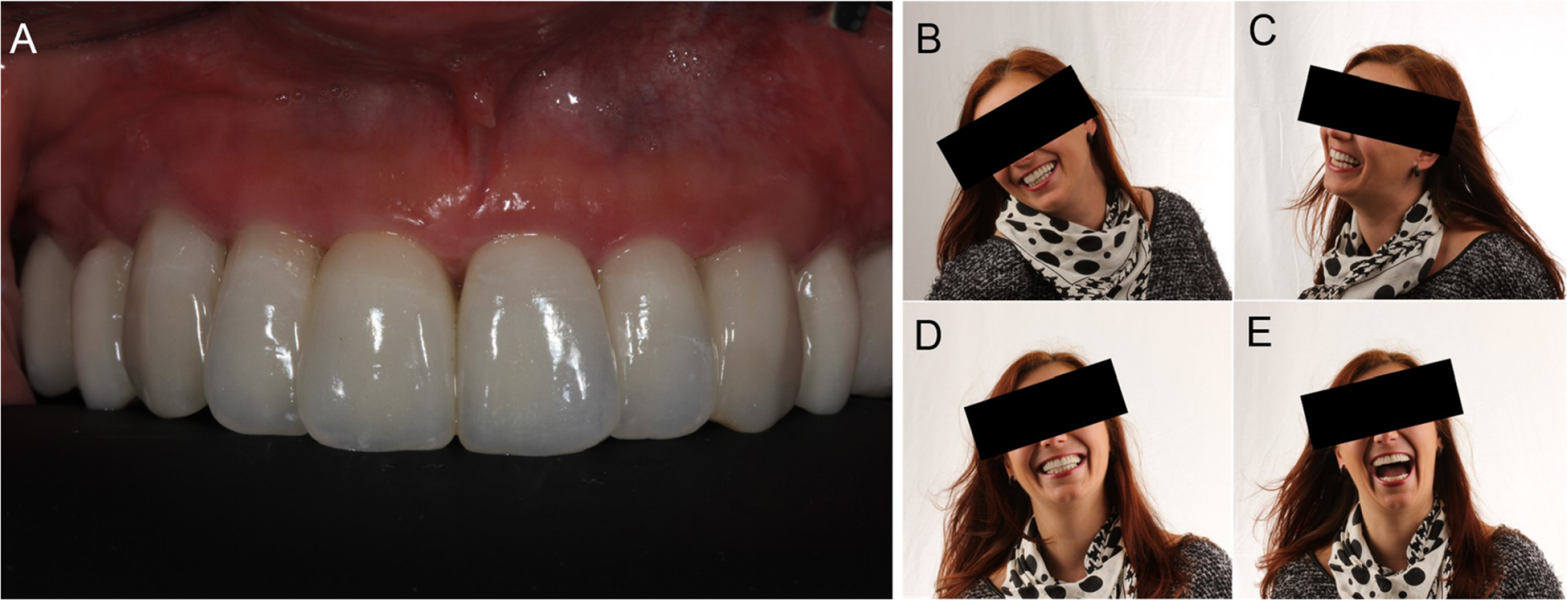
One year follow-up control. **a** Detail of the aesthetic integration. **b**, **c**, **d**, **e** Life changing dentistry: the patient is happy with complete rehabilitation, satisfactory aesthetic and functional results

The results of the patient satisfaction questionnarie are summarized in Table 5. The great majority of patients were satisfied with the treatment received (Fig. 12a, b, c, d, e) and no patients reported being dissatisfied when answering the 4 questions asked in the questionnaire.

**Table 5:** Patient satisfaction wth the treatment received

Question	Very satisfied	Satisfied	Neither satisfied nor dissatisfied	Dissatisfied	Very dissatisfied
Overall, how satisfied are you with the treatment received?	10/12 (83.3%)	2/12 (16.7%)	0/12 (0%)	0/12 (0%)	0/12 (0%)
Are you satisfied with the function of your implant-supported restorations?	12/12 (100%)	0/12 (0%)	0/12 0%	0/12 (0%)	0/12 (0%)
Are you satisfied with the esthetics of your implant-supported restorations?	11/12 (91.7%)	1/12 (8.3%)	0/12 (0%)	0/12 (0%)	0/12 (0%)
Are you satisfied with the cleanability of your implant-supported restorations?	9/12 (75%)	3/12 (25%)	0/12 (0%)	0/12 (0%)	0/12 (0%)

## Discussion

Although guided surgery has become increasingly popular and widespread, there are currently only a few studies in the literature that present the results obtained with the guided flapless placement of implants in post-extraction sockets and their immediate functional loading by means of complete fixed-arch reconstructions without artificial gum [28–31].

Most of the studies in the literature, in fact, refer to the immediate loading of *hybrid* prostheses (such as Toronto Bridges, “all-on-four,” and “all-on-six” dentures), characterized by the presence of a bar connecting the implants and, more relevant, artificial gum [22–26]. It is obvious how such prosthetic rehabilitations differ so thoroughly from a full-arch fixed prosthesis, which is characterized by the absence of artificial gum and much more delicately manageable for the clinician, from an aesthetic point of view [28–31].

In a recent clinical and radiographic study on implants placed in post-extractive and healed sites, inserted using flapless guided surgery, and immediately loaded, Ciabattoni et al. [28] have reported successful clinical results. In that study, 285 implants were installed in 32 patients with a double-guide template technique [28]; in detail, 197 implants were inserted in fresh extraction sockets (137 maxilla, 60 mandible) and 88 in healed sites (58 maxilla, 30 mandible). All implants were immediately loaded by means of fixed full-arch restorations and followed for a period of 3 years [28]. The outcome variables were implant survival, prosthesis survival, and marginal bone levels [28]. At the end of the study, a high implant survival rate (97.5%) was reported, with only 7 fixtures failed (3 in fresh extraction sockets of the maxilla, 2 in fresh extraction sockets in the mandible, and 2 in maxillary healed sites) [28]. All fixed full arches maintained stability and good functionality during the entire follow-up. Finally, the marginal bone loss accounted to 1.32 mm (± 0.41) at the 3-year follow-up control [28]. The authors concluded that flapless guided implant surgery with the double-guide template technique is a predictable treatment procedure, capable of guaranteeing predictable outcomes while decreasing length of treatment time and patient discomfort [28].

Polizzi et al. investigated the clinical and radiographic outcomes of immediate fixed restorations on maxillary implants, inserted in fresh extraction and healed sites by using the NobelGuide® system [29]. Twenty-seven patients were included in the study and were treated with flapless guided implant surgery and immediate full-arch or partial restorations. The patients were followed for a period of up to 5 years and the clinical outcomes were implant survival, marginal bone remodeling, soft-tissue parameters, and complications [29]. Among the 160 implants assessed, only four failures were reported, for a cumulative survival rate of 97.3%. At the end of the study, all prostheses were functioning. The marginal bone resorption from insertion to 2 years amounted to 0.85 mm (± 1.28); from insertion to the last radiographic control was 1.39 mm (± 1.88); and between 2 years and the last control was 0.64 mm (± 1.66) [29]. Finally, the soft-tissue response was excellent and only a few minor complications were reported [29].

In 2013, Meloni et al. [30] reported on a guided implant surgery protocol for the immediate delivery and functional loading of screw-retained provisional metal-acrylic full-arch prostheses. In total, 60 implants were placed in 10 patients: among these implants, 22 were inserted in fresh extraction sockets [30]. The final prostheses were delivered after 6 to 12 months. All patients were followed for a period of at least 1 year [30]. The outcome measures of this study were implant survival, patient satisfaction, and marginal bone loss [30]. At the end of the study, no implants were lost, for a survival rate of 100%; in addition, no complications (either biological or prosthetic) were reported [30]. All patients felt comfortable with the treatment procedures and were fully satisfied with the final functional and aesthetic result [30]. Finally, the mean marginal bone loss amounted to 1.4 mm (± 0.3) [30].

In a subsequent prospective clinical study, which represented the development of the previous one, the same researchers reported on the clinical and radiographic outcomes of 20 patients treated with guided implant surgery and immediate loading of computer-assisted-design/ computer-assisted-manufacturing (CAD/CAM) fixed full-arches [31]. In total, 120 fixtures were installed in 23 jaws, supporting immediately loaded fixed full-arch prostheses [31]. After 30 months, the implant survival rate was 97.7%, with only minor prosthetic complications encountered, a marginal bone remodeling of 1.08 mm (± 0.34), a mean probing-pocket-depth value of 2.84 mm (± 0.55), and a mean bleeding-on-probing of 4% (± 2.8%) [31]. The authors concluded that guided implant surgery and immediate functional loading of fixed full arches represent a viable option for the treatment of completely edentulous jaws [31].

The results of our present study seem to confirm the evidence emerging from the aforementioned literature [28–31]. In our present study, 12 patients received 110 implants (65 of them post-extractive), placed flapless through a guided surgery procedure and then immediately loaded by means of a provisional fixed full arch. After a provisionalization period of 6 months, 72 fixed prosthetic restorations were delivered, in order to prosthetically reconstruct the complete arch. The implant stability at placement was successful. Only two implants failed and were removed, in one single patient, for a 1-year implant survival rate of 98.2% (108/110 surviving implants). A few biologic and prosthetic complications were reported, with 8/12 prostheses that did not undergo any failure or complication during the entire follow-up period. At the 1-year follow-up control, excellent soft-tissue stability was found, and patients were satisfied with the treatment.

The advantages of our present guided surgical and prosthetic protocol can be summarized as follows. First, the flapless implant placement can reduce intra- and post-operative patient discomfort and morbidity, as indicated by different systematic reviews [20, 35] and clinical studies [36–38]. In fact, this minimally invasive approach significantly reduces the time required for surgery [36, 37]. In a controlled study, Arisan et al. demonstrated that the flapless guided surgery can significantly shorten surgical time, as compared to the conventional open-flap surgery [37]. Since the duration of the surgical intervention influences morbidity for the patient, computer-assisted flapless implant placement can reduce the incidence of surgery-related complications, such as bacteremia and infections [38]. Fortin at al. have demonstrated that the optimization of implant placement, through guided surgery, may be an option to successfully avoid having to use bone augmentation procedures [39]. But for the clinician, the main advantage in the use of guided surgery is the possibility of planning the implants (at the requisite position, inclination, and depth) in the best possible way, avoiding dangerous anatomical structures (inferior alveolar nerve and maxillary sinus) and taking into account the emergence profiles and the overlying prosthesis. It is the so-called “prosthetically guided” placement, where the implants are planned in the best possible way to support the prosthesis, which reduces the need of prosthetic compromises. In fact, during the planning of the fixtures in the software, the prosthetic suprastructure is imported and included; hence, the optimal position of the implants (depth, mesio-distal, bucco-lingual as well as inclination) in relation to the future prosthetic rehabilitation can be obtained. As such, the fixtures can be planned to support a prosthesis that provides the functional, biologic, and aesthetic requirements and at the same time the anatomy of the bone as well as prosthetic boundaries can be taken into account. In the present study, care was taken to plan the implants in the best position, depth, and inclination, in order to engage the fixtures as much as possible in the residual bone (exceeding the apex of fresh extraction sockets at least 3–4 mm) to maximize stability, and at a distance of 2–3 mm from the residual buccal bone walls, to avoid aesthetic problems. At the same time, however, the planning was prosthetically guided, with the axis of the implants that had to be, as much as possible, in the center of the teeth. The final planning was the result of a compromise between the residual amount of bone available, and the ideal prosthetic emergence profile. This is not a trivial matter, particularly when a fixed full arch without artificial gum is planned; in fact, with guided implant surgery, the improved accuracy in implant placement can provide a better platform for the final prosthetic restoration. Immediately after surgery, the pre-fabricated provisional full arch can be delivered to patients and loaded, improving patient satisfaction, comfort, and treatment acceptance, without the need to use any removable denture. The application of these pre-fabricated restorations results in acceptable success rates, as reported in the literature. In addition, the immediately loaded provisional restoration may guide the soft-tissue healing for an optimal aesthetic result with minimal recession, taking advantage also of the extraction socket healing potential.

In 2015, Furhauser et al. [40] published a clinical study in which stereolithographic surgical guides were used to insert single-tooth implants for the replacement of upper incisors. The authors evaluated the accuracy in implant placement and the aesthetic result by means of the pink aesthetic score (PES) after a mean follow-up of 2.3 years [40]. Even though guided, a mean deviation from the originally planned position of 0.84 mm (measured at the implant shoulder) was found [40]. However, the authors found that deviations ≥0.8 mm resulted in significantly worse aesthetic results (median PES: 9.5) than with more accurate implant positions (median PES: 13, *p* = 0.039) [40].

The issue of accuracy in transferring the implant position from the software to the surgery actually remains to be solved, as demonstrated by various studies [41, 42] and literature reviews [14–16, 20, 35]. Therefore, considering that these deviations may depend on several factors that cannot be completely controlled [14–16, 35, 41, 42], it is advisable for clinicians to carefully select patients as candidates for guided surgery, especially at the beginning of their own learning path of the technique.

In the coming years, the continuous technological advancements in the world of digital dentistry, together with improvements in devices such as scanners [43, 44] and software for planning and in the manufacturing of surgical guides [45], might actually reduce the inaccuracy of guided implant placement.

Moreover, in a recent systematic review of flapless guided surgery procedures, the authors have found excellent survival rates (97.2%) in the included studies and minimal mean marginal bone loss (1.45 mm) during 1–4 years of follow-up [20]; however, complications were also found, such as insufficient primary implant stability at placement in the fresh extraction sockets, as well as occasional surgical guide fractures or fractures of the immediate provisional restoration [20]. Once again, the authors concluded that there is a learning curve to achieve complete treatment success; certainly, the selection of the case is important and it would be advisable to start with simpler cases, then gradually learning how to deal with more complex cases [20].

Finally, the present study has certain limits, such as the limited number of patients enrolled and the short follow-up time. In addition, the retrospective is not the most suitable study design for obtaining indisputable scientific data [46, 47]. Moreover, this study was performed following a mixed, digital-analog workflow and this could be considered as another limitation of the present research, since the use of intraoral scanners is today well established [44] and could potentially reduce the number of steps and procedures described here. Therefore, new studies should be conducted with a prospective design and possibly randomized controlled trials, in order to draw more specific conclusions on the validity and effectiveness of this technique.

## Conclusions

In our present study, 12 patients received 110 implants (65 of them post-extractive), placed flapless through a guided surgery procedure and then immediately loaded by means of a provisional fixed full arch. After a provisionalization period of 6 months, 72 fixed zirconia-ceramic prosthetic restorations (53 single crowns, 17 bridges, and 2 fixed full arches) were delivered. The results showed a 1-year implant survival rate of 98.2% (108/110 surviving implants) and good soft-tissue stability.

Our present surgical and prosthetic approach presents several advantages. First, only one surgical session is required for tooth extraction, implantation and application of provisional prosthesis. For the patients social life, this concept allows a reduction of discomfort and facilite their return to professional life. For the dental rehabilitation, provisional restoration guides the soft-tissue healing for an optimal aesthetic result. Within the limitations of this study, combining a CBCT-derived surgical guide to an immediate implant placement in post-extraction sockets together with immediate provisionalization and loading seems to be a safe and predictable therapy, with high survival rates and excellent aesthetic results, when applied in indicated cases. Further studies on larger samples of patients and with longer follow-up controls are needed to draw more specific conclusions about the long-term results with the present technique.

## Data Availability

The datasets used and/or analysed during the current study are available from the corresponding author on reasonable request.

## Abbreviations

3D: three-dimensional
BOP: Bleeding on probing
CBCT: Cone beam computed tomography
CT: computerized tomography
DICOM: digital imaging and communication in medicine
PES: pink aesthetic score
PPD: Probing pocket depth
